# Analysis of warpage and reliability of very thin profile fine pitch ball grid array

**DOI:** 10.1016/j.heliyon.2024.e35459

**Published:** 2024-07-30

**Authors:** Chin-Hsin Lo, Te-Yuan Chang, Ting-Yu Lee, Sheng-Jye Hwang

**Affiliations:** Department of Mechanical Engineering, National Cheng Kung University, Tainan, Taiwan

**Keywords:** Advanced IC packaging, Residual stress, Redistribution layer, Pressure–volume–temperature–cure equations, Viscoelasticity, Reliability

## Abstract

With the evolution of advanced integrated circuit (IC) packaging technology, the use of experiments to identify package performance and life expectation will take a significant amount of time and cost to finish the job. To reduce the cost of research and testing, predictive analyses of reliability and performance using simulation tools have become a feasible approach for the IC assembly industry. Therefore, this study utilized Moldex3D molding simulation software to analyze very thin profile fine pitch ball grid array (VFBGA) packages and established a numerical analysis procedure from the molding and curing process, the post-mold cure (PMC) process, to a thermal cycling test (TCT) to predict the amount of package warpage during processing and reliability after TCT.

The results showed that the warpage trends of both experiments and simulations during the same temperature ramping process were similar. In the thermal cycling analysis, potential failure locations were found to be at the copper pillars and redistribution layer (RDL), where the maximum Von Mises stress occurred at the lowest temperature (−65 °C). The fatigue life model, Coffin–Manson model, was used to calculate the potential fatigue life at the two locations, resulting in 1689 cycles (copper pillars) and 9706 cycles (RDL L1).

## 1 Introduction

Recent studies have shown that the cure-induced shrinkage of epoxy molding compound (EMC) is also one of the significant factors affecting the warpage of the integrated circuit (IC) package, in addition to the thermal mismatch induced by the coefficient of thermal expansion (CTE) inconsistency between parts. Kelly et al. [1] recommended that considering thermal and chemical shrinkage will enhance the accuracy of warpage analysis. Sato and Yokoi [2] and Rimdusit and Ishida [3] observed the flow behavior of EMC through experiments and established the relationship of cure degree and viscosity with temperature and time, respectively. Bidstrup-Allen et al. [4] used Kamal's model to simulate the change in the curing rate with the reaction time during the EMC polymerization process and applied the Castro–Macosko model to describe the change in viscosity at different degrees of cure. Hu et al. [5] found that there is a cure shrinkage in the cross-linking process of the EMC occurring at high temperatures, and it is mentioned that the amount of warpage can be accurately predicted by taking into consideration the elasticity properties related to temperature, the coefficient of thermal expansion, and the chemical shrinkage at the same time.

Chang and Hwang [6] observed that the cure reaction has a certain degree of influence on pressure-volume-temperature equations (*P–V–T*). Therefore, a mathematical model of pressure-volume-temperature-cure equations (*P*–*V*–*T*–*C*) was established by Chang and Hwang [7] to describe the effect of pressure, temperature, and degree of cure on the volume change of EMC. The applicability of the *P*–*V*–*T*–*C* relation model is also confirmed by the studies conducted by Teng [8], Deng [9], and Wang [10]. Their study results showed that considering the chemical shrinkage of EMC during the curing process, combining with the thermal shrinkage, can increase the simulation accuracy. Polymers have viscoelastic properties and slowly deform or change over time under fixed stress. Amagai [11] stated that most packaging plastics exhibit viscoelastic behavior even at room temperature. Amagai [11], Park et al. [12], Yeung [13], and Wang [14] showed that the viscoelastic model of nonlinear EMC materials described by the generalized Maxwell model can predict the warpage and stress distribution more accurately than the linear elastic material model. Shue et al. [15], Lin et al. [16], and Wang [17] obtained the equations for the relationship between the degree of cure and the stress relaxation by measuring the loss modulus and the storage modulus of EMC under different cure conditions, which were defined as the cure shift factor (CSF). Then, the Williams–Landel–Ferry equation (WLF) defining the temperature shift factor was considered, and the total shift factor in the generalized Maxwell model was proposed to be the product of the temperature and cure shift factors. Loh et al. [18], Guo [19], and Lee et al. [20] verified that the analysis method considering the packaging process (mold flow, curing, and post-curing) can help to increase the accuracy of the prediction results.

In recent years, one of the feasible methods is to conduct the accelerated life test in the computer aided engineering (CAE) software and then, use the suitable fatigue life model to convert the analysis results into reliability information. Common fatigue models are mainly divided into strain energy, plastic strain, and creep strain, which can be obtained by simulation. Among these models, the plastic strain was first proposed by Coffin and Manson, called the Coffin–Manson fatigue life model [21]. The concept of this model uses the plastic strain of the material as the measurement factor, and the low-cycle fatigue (LCF) times are calculated through the plastic deformation amplitude obtained by the thermal cycle. On the basis of the Coffin–Manson model, Engelmaier [[22], [23], [24]] proposed a lead-containing solder fatigue model considering parameters such as cycle frequency and solder ball average temperature, called the Engelmaier model. Singh et al. [25] conducted a reliability analysis for large ultra-thin glass ball grid array (BGA) packages.

In addition to solder balls, redistribution layer (RDL) is one of the reliability targets. Shie et al. [26] studied the failure mechanism of Cu–Cu pillars using a thermal cycle test. The results of experiments and finite element analysis (FEA) showed that the maximum stress is located at the connection between the RDL and the copper pillars, where the bonding strength affects the reliability of components. Che et al. [27] simulated the JEDEC thermal cycle test on the copper interconnection in the bumpless flip chip package (BFCP). The stress–strain curve of the copper material is described by the multilinear kinematic hardening constitutive equation, and the Coffin–Manson model to calculate the effect of plastic strain on fatigue life. Lin [28] analyzed whether the residual stress considered in the thermal cycling test (TCT) simulation has a significant difference in the reliability results.

This study utilizes numerical simulation software to research the filling process of very thin profile fine pitch ball grid array (VFBGA) IC packaging and its reliability analysis. The product reliability is predicted based on the simulation results presented through visualization. Furthermore, the validation of the simulation results and experiment results is conducted to confirm the accuracy of the reliability predictions. In this study, EMC material properties are modelled in dependency of the cross Castro–Macosko viscosity model, Kamal's cure kinetics model, the *P*–*V*–*T*–*C* relationship, and the generalized Maxwell model. In the reliability analysis, the residual stress in the post-mold cure (PMC) process was considered, and the fatigue life model was the Coffin–Manson model.

## 2 Theory

The EMC material used below is provided by the customers of our project sponsor -- Advanced Semiconductor Engineering Inc. located in Kaohsiung, Taiwan.

### 2.1 Viscosity model

**Fig. 1:**
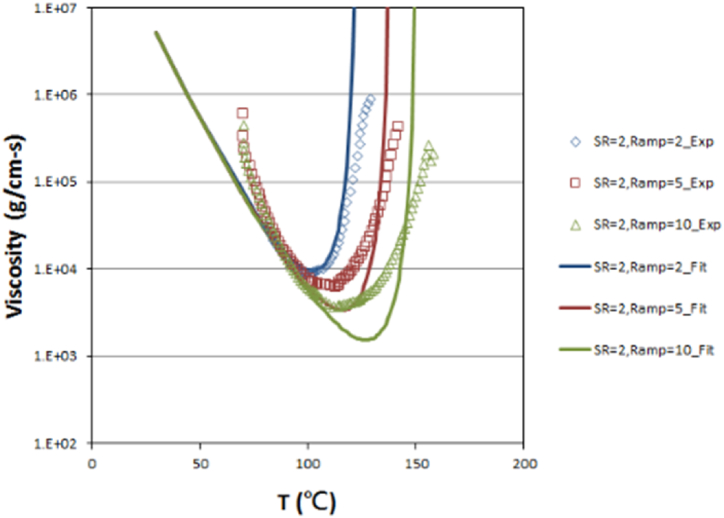
Relationship curve of viscosity and temperature at shear rate 2 (1/sec).

The viscosity characteristics of EMC are complex. During the filling process, the viscosity of EMC decreases first due to B-stage reaction and then increases due to cross-linking reactions. The viscosity behavior of EMC is measured with an Anton Paar MCR 502 Rheometer and fitted with cross Castro–Macosko viscosity model by the laboratory of CoreTech System Co., Ltd. (Moldex3D) located in Hsinchu, Taiwan. In the rheometer experiment, the EMC is placed between parallel plates and a fixed angular frequency is applied to the upper plate for rotation. The viscosity is obtained by observing the changes in shear strain rate between the upper and lower parallel plates of the EMC. Fig. 1 illustrates the relationship between viscosity and temperature under different rates of temperature increase (Ramp in Fig. 1 below).

(1) $$\eta =\frac{\eta_{0}{\left(\frac{C_{g}}{C_{g}‐C}\right)}^{b_{1}+b_{2}C}}{1+{\left(\frac{\eta_{0}‐\dot{\gamma }}{\tau^{*}}\right)}^{1‐n}}$$

(2) $$\eta_{0\;}=A∙\exp\left(\frac{T_{b}}{T}\right)$$

*C*: degree of cure; *η*: viscosity; *η*_0_: zero-shear-rate viscosity; $\dot{\gamma }$: shear strain rate; τ*: critical shear stress; *n*: power law index; *C*_*g*_: degree of cure at gel point; *b*_1_, *b*_2_, *A*, *T*_*b*_: model constants; *E*_*η*_: activation energy; *R*: ideal gas constant.

### 2.2 Cure kinetics model

**Fig. 2:**
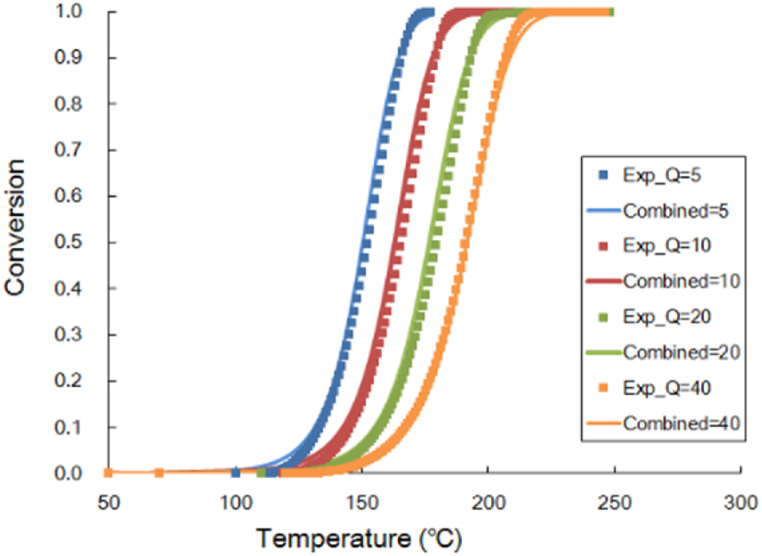
Relationship curve of conversion and temperature scanning from 30 to 250 °C with several heating rates: 5,10, 20, 40, °C/min.

When EMC is heated through B and C stages of cure reaction, cross-linking occurs. Kamal's cure kinetics model is used to describe the curing behavior of EMC in this study. The cure behavior is measured with a PerkinElmer DSC 8500 differential scanning calorimetry (DSC) and fitted with Kamal's cure kinetics model by the laboratory of CoreTech System Co., Ltd. (Moldex3D) located in Hsinchu, Taiwan. In the DSC experiment, the EMC material is divided into two groups and placed in different heating systems. When the test group undergoes phase change or chemical change, endothermic and exothermic phenomena occur, causing the temperature to differ from the control group. Both groups achieve the same temperature through conducting the input and output energy of the control group. Then calculate the input or output energy to obtain the curing relationship curve between endothermic and exothermic processes and time. Kamal's cure kinetics model was used to describe the cure behavior of EMC in this study. Fig. 2 represents the schematic curve from the DSC measurement, showing the heat absorption curve of the material as temperature increases under different rates of temperature increase (Q in Fig. 2 below).

(3) $$\dot{C}=\frac{dC}{dt}=\left(K_{a}+K_{b}∙C^{m}\right)∙\left(1‐C\right)n$$

(4) $$K_{a}=A∙\exp\left(‐\frac{T_{a}}{T}\right)$$

(5) $$K_{b}=B∙\exp\left(‐\frac{T_{b}}{T}\right)$$

$\dot{C}$: cure reaction rate; *m*, *n*: model constants; *A*, *B*: cure reaction frequency factor; *K*_*a*_, *K*_*b*_: cure reaction rate constant; *T*_*a*_, *T*_*b*_: activation temperature.

### 2.3 *P–V–T–C* model

**Fig. 3:**
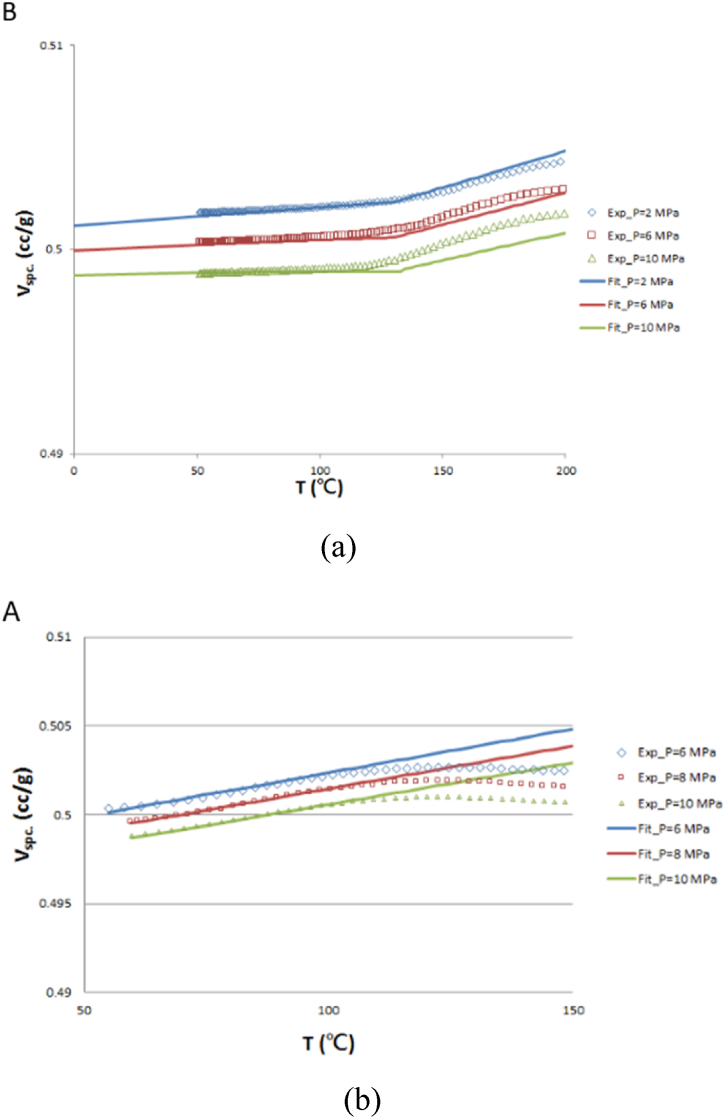
*P*–*V*–*T*–*C* relationship: (a) fully cured, (b) uncured.

The *P*–*V*–*T*–*C* model is used to model the specific volume change caused by CTE and chemical shrinkage after the packaging process. The curves are plotted based on data measured with U-CAN PT-6800 *P*–*V*–*T*–*C* instrument and fitted with two-domain modified Tait model by the laboratory of CoreTech System Co., Ltd. (Moldex3D) located in Hsinchu, Taiwan. In the *P*–*V*-*T*-*C* experiment, curing tests are conducted under various temperature and pressure conditions to establish the relationship between the degree of cure and the specific volume of EMC under different temperature and pressure conditions. Fig. 3(a) illustrates the *P*–*V*-*T*-*C* behavior of EMC under a fully cured state. The inflection point of the curve in Fig. 3(a) corresponds to the *T*_*g*_ point (glass transition temperature) of fully cured EMC. On the other hand, Fig. 3(b) illustrates the *P*–*V*-*T*-*C* behavior of EMC under an uncured state. The experiment data are obtained through incremental temperature measurements. Thermal expansion due to heating causes an increase in the specific volume occurring through the whole stage of the data curve, while the major curing shrinkage due to the curing process causes a decrease in the specific volume occurring in the later stage of the data curve. Consequently, the inflection point of the data fitting curve in Fig. 3(b) is not the *T*_*g*_ point of the uncured EMC. The fitting data of the uncured EMC are obtained below the *T*_*g*_ point of the fully cured EMC with temperature measurements, while the values above the *T*_*g*_ point are obtained by extrapolation.

(6) $$\frac{1}{V}=\frac{1}{V_{uncured}}∙\left(1‐C\right)+\frac{1}{V_{cured}}∙C$$

(7) $$V_{uncured/cured}=V_{0}\left[1‐\alpha ∙\ln\left(1+\frac{P}{B_{p}}\right)\right]$$

(8) $$V_{0}=\left\{ \begin{array}{ll} b_{1S}+b_{2S}\left(T-b_{5}\right), & \text{if}\;T\leq T_{trans}\\ b_{1L}+b_{2L}\left(T-b_{5}\right), & \text{if}\;T\;>\;T_{trans} \end{array}\right.$$

(9) $$B_{p}=\left\{ \begin{array}{ll} b_{3S}∙\exp\left[-b_{4S}∙\left(T-b_{5}\right)\right], & \text{if}\;T\leq T_{trans}\\ b_{3L}∙\exp\left[-b_{4L}∙\left(T-b_{5}\right)\right], & \text{if}\;T\;>\;T_{trans} \end{array}\right.$$

(10) $$T_{trans}\left(P\right)=b_{5}+b_{6}∙P,\alpha =0.0894$$

C: degree of cure; *V*: specific volume; *V*_0_: specific volume at zero-gauge pressure; *α*: universal constant; *P*: pressure; *B*_*p*_: pressure sensitivity of the material; *b*_1*S*_, *b*_1*L*_, *b*_2*S*_, *b*_2*L*_: coefficient of linear change in specific volume with temperature; *b*_3*S*_, *b*_3*L*_, *b*_4*S*_, *b*_4*L*_: material constants; *b*_6_: linear increase in *T*_*trans*_ with pressure; *b*_5_: transition temperature at zero-gauge pressure.

### 2.4 Viscoelastic model

**Fig. 4:**
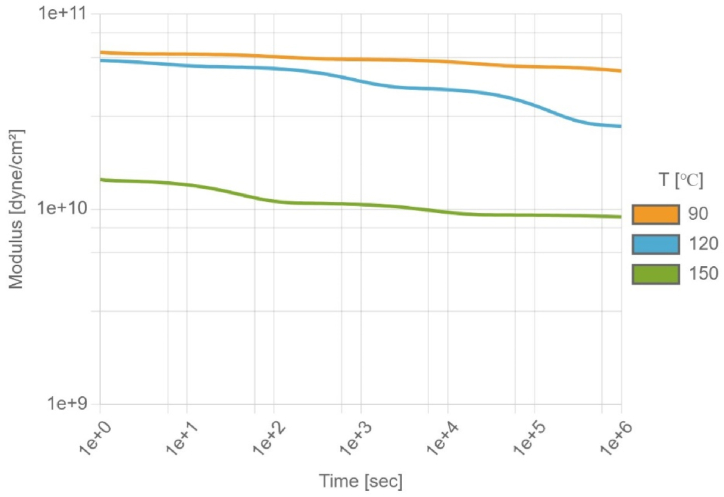
Relationship between elastic modulus and time at different temperatures.

The dual shift factor viscoelastic model is used to describe the viscoelastic behavior of EMC in this study. The curves are plotted based on data measured with a PerkinElmer Diamond dynamic mechanical analyzer (DMA) and fitted with the dual shift factor model by the laboratory of CoreTech System Co., Ltd. (Moldex3D) located in Hsinchu, Taiwan. In DMA experiments, periodic stress (sinusoidal stress) is applied to the material, and the corresponding strain response is measured. This is done to obtain dynamic mechanical properties such as storage modulus, loss modulus, etc. The data measured by DMA were converted from frequency-temperature to time-temperature. Finally, the time-temperature superposition principle (TTS) [29] is applied to get the relaxation master curve for long timescales. For polymers, due to time dependence and nonlinearity under different temperatures, their moduli are not constants, as illustrated in Fig. 4. Since the main ingredient of EMC is epoxy which is a thermosetting polymer, its viscoelastic behavior will depend not only on temperature but also on degree of cure. Thus, the dual shift factor model with temperature and degree of cure shift factors are included in the model which can be used to describe the post-mold cure behavior of packaging process [30].

(11) $$\lambda_{i}\left(T,C\right)=\lambda_{i}\left(T_{f}\right)∙a_{T}\left(T\right)∙a_{C}\left(C\right)$$

(12) $$a_{T\;}=\left\{ \begin{array}{c} \exp\left(\frac{‐A_{1}\left(T‐T_{f}\right)}{A_{2}+\left(T‐T_{f}\right)}\right),\text{for}\;T\geq T^{*}\\ \exp\left(\frac{\Delta H_{T}}{R}\left(\frac{1}{T}-\frac{1}{T_{0}}\right)\right),\text{for}\;T\;<\;T^{*} \end{array}\right.$$

(13) $$a_{C\;}=\exp\left(‐\frac{\Delta H_{C}}{R}\left(\frac{1}{T_{g}\left(C\right)}‐\frac{1}{T_{g1}}\right)\right)$$

(14) $$T_{g}\left(C\right)=T_{g0}+\frac{\gamma C\left(T_{g1}‐T_{g0}\right)}{1‐\left(1‐\gamma \right)C}$$

*λ*_*i*_(*T*, *C*): relaxation time relative to temperature and curing degree; *a*_*T*_(*T*): shift factor of temperature; *a*_*C*_(*C*): shift factor of curing degree; $T_{f}$: reference temperature; $T^{*}$: critical temperature; A1, A2: WLF model constants; ΔHT/*R*, ΔHC/*R:* active energy; Tg: glass transition temperature; *R*: ideal gas constant.

## 3 Mold flow and warpage analysis

**Fig. 5:**
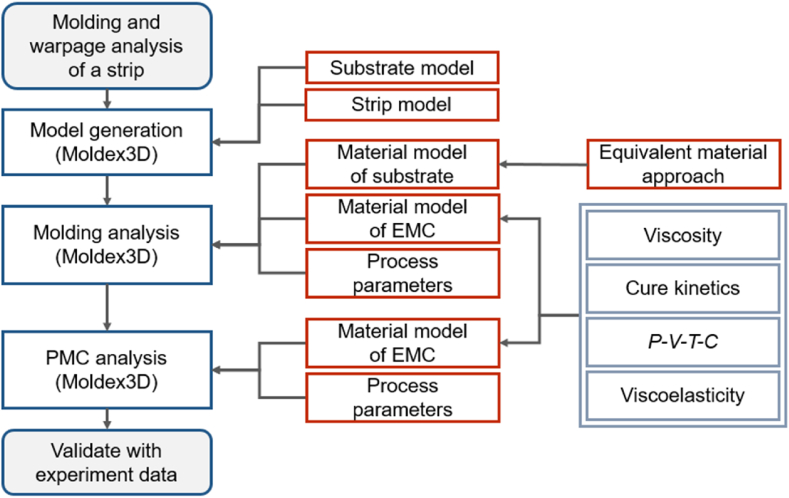
Mold flow and warpage analysis flow chart.

The modeling and mold flow analysis of the strip model all use Moldex3D commercial software, which is a CAE mold flow software used in the plastic injection molding industry and has real 3D simulation analysis technology. The flow chart of mold flow and post-mold cure analysis is shown in Fig. 5. In the analysis of mold flow and post-mold cure, it is necessary to first establish a model of the whole strip, including all substrate layers. Then, input the measured material parameters and process parameters to conduct the simulation. The equivalent method is used to describe the material properties of the RDL layers in the substrate since the RDL layers are composite material layers. Finally, the simulations are conducted, and the simulation results are validated against the experiment results.

### 3.1 Geometry

**Fig. 6:**
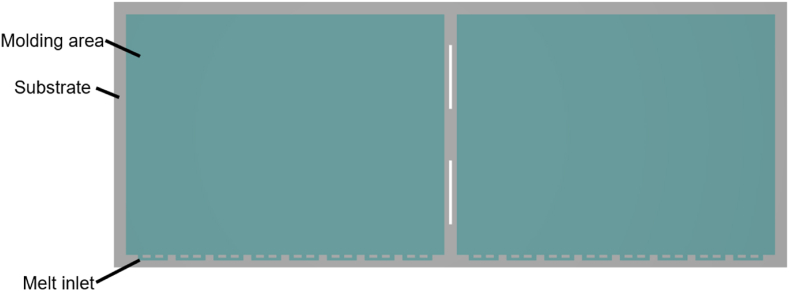
Top view of a strip.

**Fig. 7:**
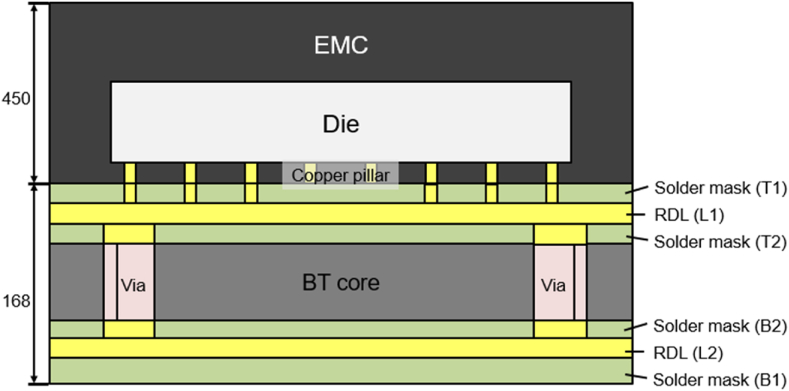
Detailed schematic cross-sectional view of the package.

In this study, the IC package in the form of a VFBGA package was used. The geometric structure design drawing was provided by Advanced Semiconductor Engineering Inc. located in Kaohsiung, Taiwan. The molding stage was packaged with a long strip. The geometry of a strip is shown in Fig. 6. Fig. 7 shows the detailed schematic cross-sectional view of the package. The thickness of the substrate and molded layer are 0.17 mm and 0.45 mm respectively.

### 3.2 Mesh model

To ensure the accuracy of mold flow and warpage trend results, the solid mesh model was established in the Moldex3D studio software by using the strip size. The mesh information of the strip model is shown in Table 1. Moreover, it was necessary to consider the hardware computational capabilities and analysis efficiency, so that the analysis time could be reduced and the results met expectations. It was assumed that.

1. The copper pillar layer was a homogeneous equivalent material layer.
2. The RDL layer was set as a homogeneous equivalent layer.
3. The solder balls, vias, and tooling holes were ignored.

**Table 1:** Mesh information of strip model.

Mesh size (μm)	300
No. of elements	6,313,284

### 3.3 Warpage analysis with equivalent model

The composite properties of both layers were calculated using the volume percentage method [31,32]:

(15) $$\delta_{eq}=\delta_{A}V_{A}+\delta_{B}V_{B}$$

**Fig. 8:**
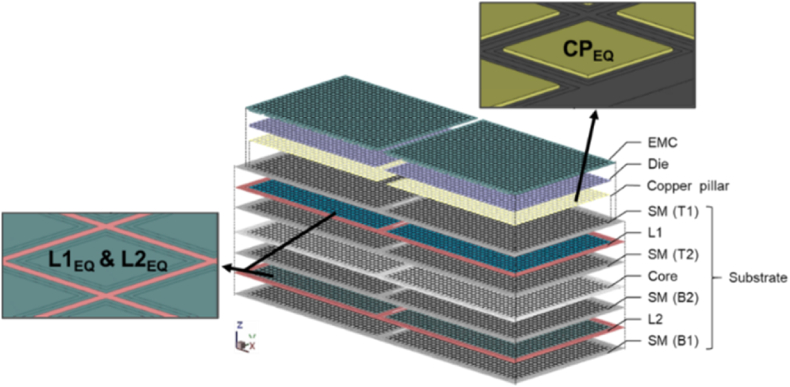
Strip model using equivalent material region.

*δ*_*eq*_: equivalent material properties of composite materials; they can be substituted for Young's modulus, coefficient of thermal expansion, Poisson ratio, density, etc.; *δ*_*A*_, *δ*_*B*_: material properties of materials A and B, respectively; *V*_*A*_, *V*_*B*_: volume percentage of materials A and B in the composite, respectively. Fig. 8 displays an exploded schematic diagram of the mesh for each layer of the substrate. The area covered by RDL included L1 and L2, which were defined as L1_EQ_ and L2_EQ_ respectively, with the length and width of a unit as the equivalent area range. The main material was copper and solder resist, and the copper volume percentage was 69 % and 66 % in L1 and L2, respectively.

### 3.4 Mold flow and warpage analysis

**Fig. 9:**
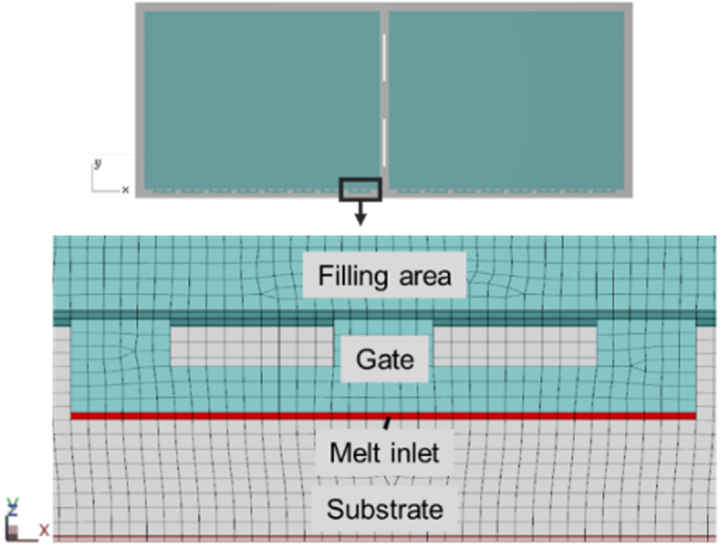
Boundary condition setting.

The mold flow analysis in Moldex3D considered the manufacturing process including material preparation, preheating, filling, in-mold cure, demolding, and cooling, each stage of the process had a corresponding temperature and time. This study used the same transfer molding package as the actual process, and the Moldex3D software provided parameter settings that had to be considered in the process (Table 2). Fig. 9 shows the melt inlet which was located on the side of the gate. Gravity was set to −980.66 cm/s^2^ in the z-direction.

**Table 2:** Process parameter settings in Moldex3D.

Parameter settings	Value	Unit
Mold temperature	80	°C
Resin temperature	185	°C
Transfer time	10	s
Transfer pressure	6	MPa
Curing time	100	s
Curing pressure	7	MPa
Curing switch	98	%
Initial conversion	0.2	%

**Fig. 10:**
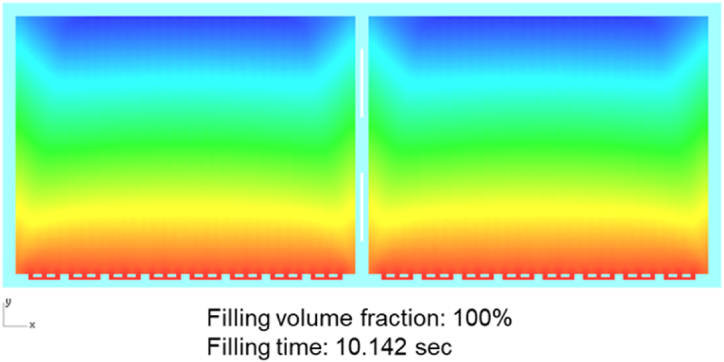
Melting front results (100 %).

The analysis results shown in Fig. 10 showed that the flow front of EMC injected into the mold cavity was considerably uniform under the uniform arrangement. It was clearly observed that the flow front near the edge of the cavity was larger than the distance between the die and the flow section on both sides of the filling area, and the flow front was formed ahead of the middle area of the cavity. The melt front filling the middle area was relatively smooth and consistent, and this phenomenon continued until the filling was 100 % complete.

**Fig. 11:**
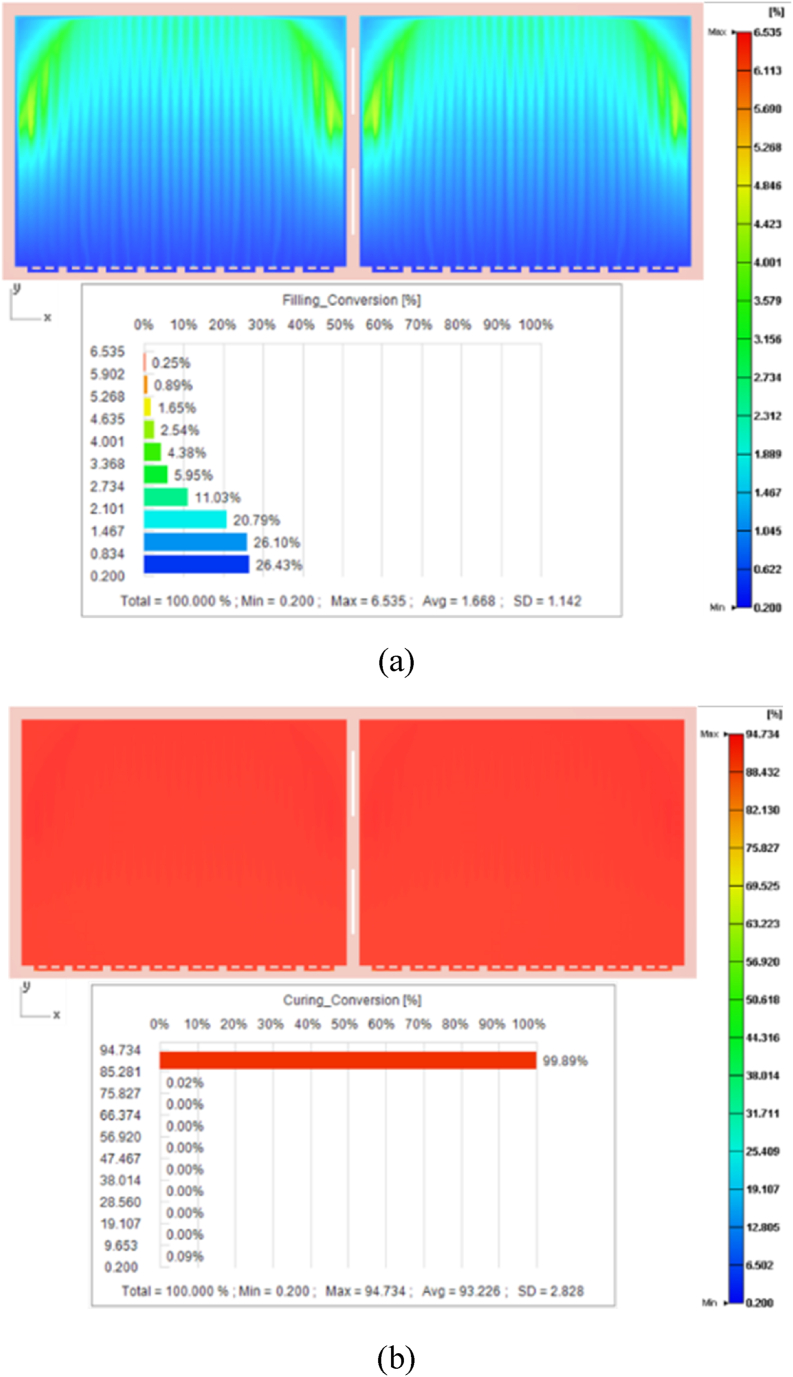
Distribution of the cure degree after (a) filling process and (b) curing process.

After the filling process, the area of cure degree near the gate was approximately 0.2 %, while the maximum cure degree at the end of the filling area was 6.54 % because of the longer heating time (Fig. 11(a)). After 100 s of 185 °C high-temperature curing stage, 99.89 % of the EMC reached a curing degree of 94.73 % (Fig. 11(b)).

**Fig. 12:**
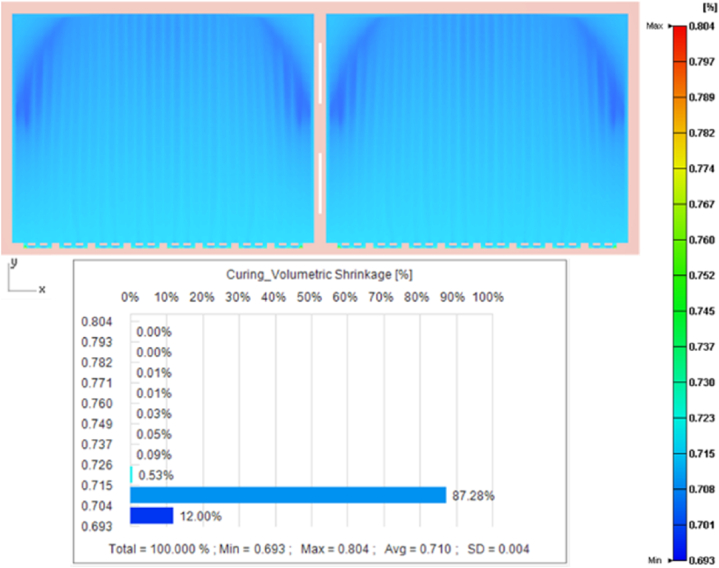
Volume shrinkage and distribution after the curing stage.

The uneven pressure and temperature distribution in the EMC molding process caused uneven volume shrinkage, resulting in residual stress and warping of the packaging. When cooling from a high temperature and high pressure to room temperature and ambient pressure, the volume shrinkage of EMC was affected by *P*–*V*–*T*–*C*. The increased shrinkage after the curing process is shown in Fig. 12.

**Fig. 13:**
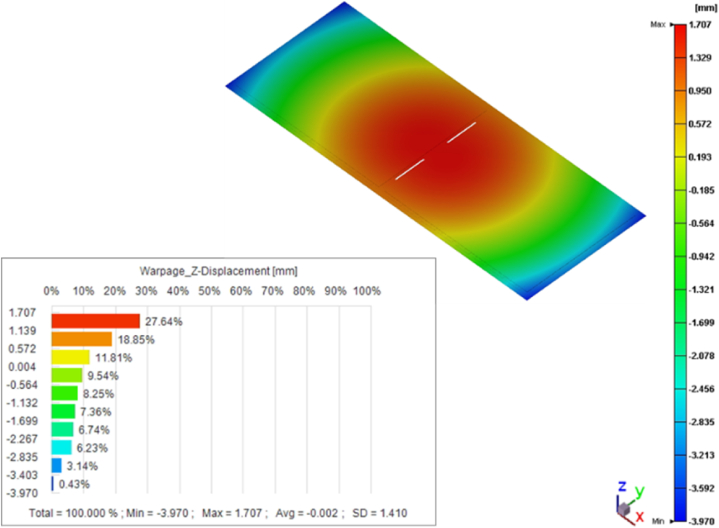
Warpage results after demolding.

After the curing stage, the packaging was taken out from the mold, and it entered the cooling stage. The warpage was described by the chemical shrinkage and the CTE mismatch of the *P*–*V*–*T*–*C* model. The analysis results are shown in Fig. 13.

## 4 Post-mold cure (PMC) analysis

**Fig. 14:**
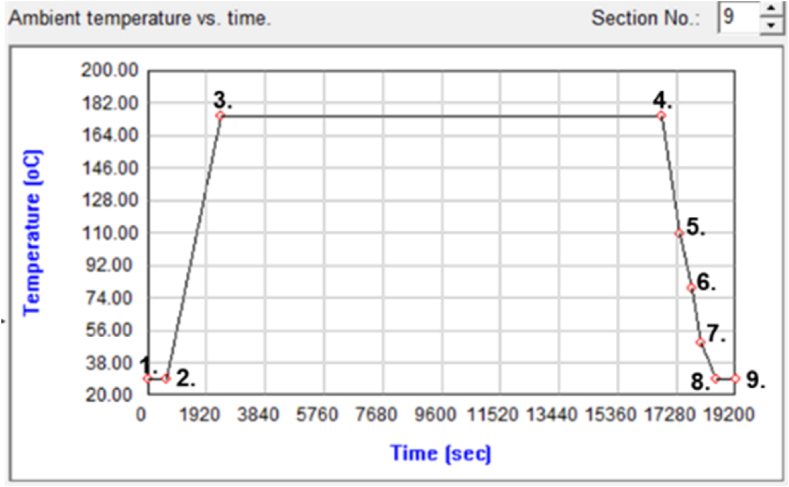
PMC process ambient condition settings.

The purpose of the post-mold cure process was to bake the uncured EMC so that the EMC curing degree reached 100 %, which could not only improve the mechanical properties and reliability of the product but also release the internal residual stress. Fig. 14 shows the temperature settings for the post-mold cure process, which were the same as the actual process conditions and those provided by our project sponsor -- Advanced Semiconductor Engineering Inc. located in Kaohsiung, Taiwan.

**Fig. 15:**
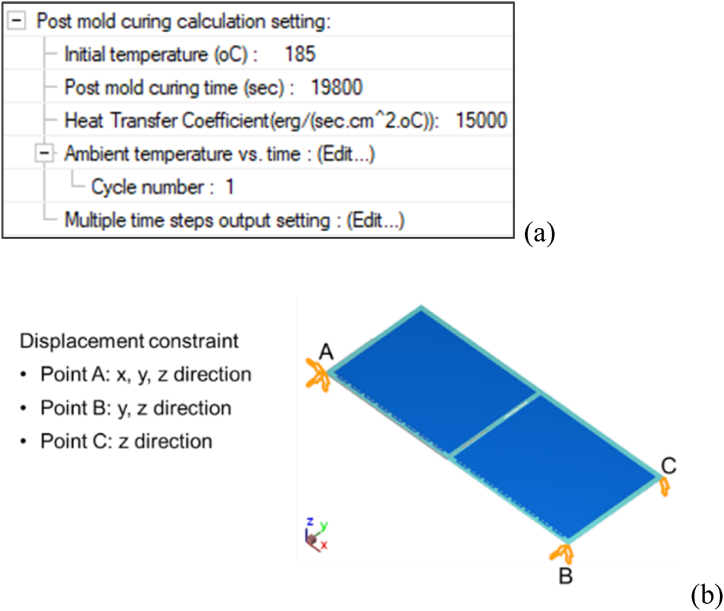
The setting of boundary conditions for post-mold cure: (a) initial temperature and heat convection coefficient, and (b) displacement constraint.

With the mold temperature of 185 °C as the reference temperature (stress-free state), the thermal convection coefficient was set to 15 W/m^2^K, as shown in Fig. 15(a); the displacement constraint was set in the point form at the three endpoints at the bottom of the substrate, as shown in Fig. 15(b).

**Fig. 16:**
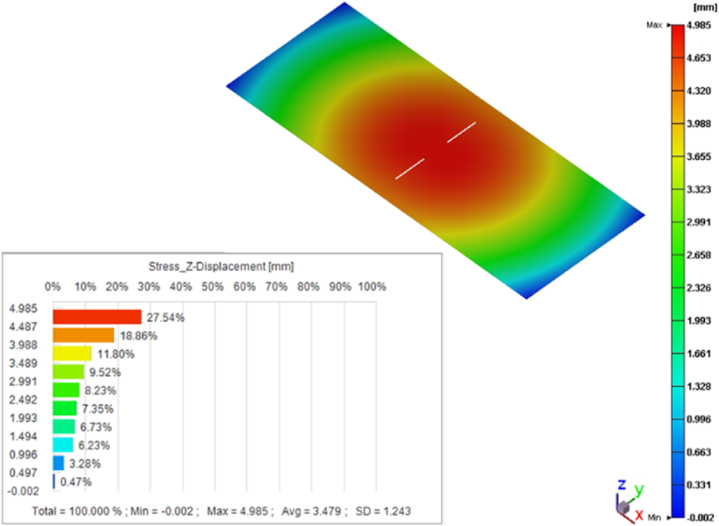
Warpage after post-mold cure process.

The long-term high-temperature environment made the viscoelastic properties of polymer materials become obvious [33]. With the participation of viscous properties, the stress relaxation of elastic solids affected the degree of deformation of EMC. Fig. 16 shows that the warping trend was similar to that before the post-mold cure process, showing a double egg yolk concave downward, while the maximum deformation exhibited a slight decrease, from 5.7 mm to 5.0 mm.

**Fig. 17:**
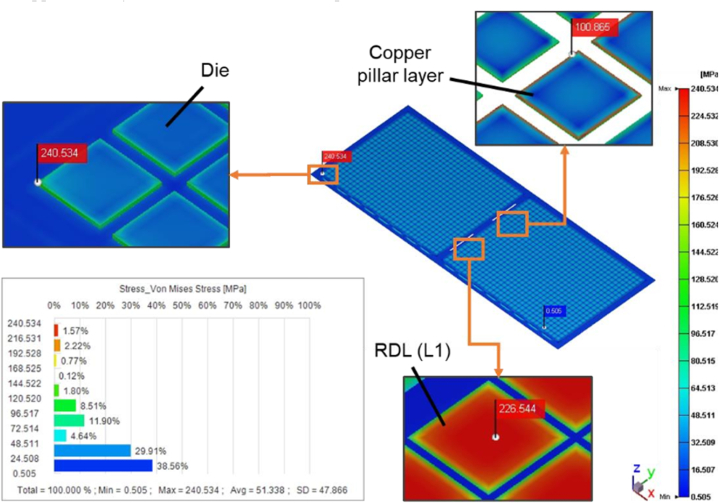
The von Mises stress results from the post-mold cure simulation.

**Fig. 18:**
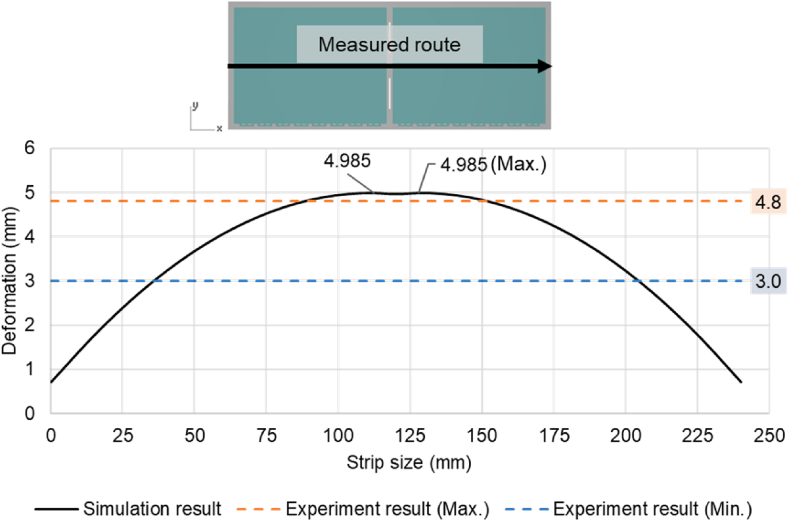
Results of the experiment after PMC.

The product of the experiment was provided by Advanced Semiconductor Engineering Inc. located in Kaohsiung, Taiwan, and the measurement temperature was approximately 30 °C at room temperature. Compared with the warpage trend of the experiment (Fig. 18) and the simulation (Fig. 16), it was observed that the warpage degree of the short side of the strip was not obvious, and the overall warpage trend was mainly on the long side. Another observation was that the warpage tendency of the filling areas of the EMC was concave downward, exhibiting the shape of a double egg yolk. After the post-mold cure process, the location with the maximum von Mises stress in the whole strip is at the corner of the die, with a value of 240.5 MPa. The maximum von Mises stress in RDL L1 is 226.5 MPa, and the maximum von Mises stress in the copper pillar is 100.9 MPa. The von Mises stress results from the post-mold cure simulation are shown in Fig. 17 below. Due to the uncertainty of human operations in manufacturing process, and measurement uncertainty in the measuring process, slight variations or errors can occur in the measured data with each measurement. Therefore, the measured warpage fell between 3.0 mm and 4.8 mm. The maximum warpage value of the post-mold cure simulation was 5.0 mm, and Fig. 18 shows that the warpage result of the simulation analysis was approximately 3.9 % different from the experimental measurement value.

## 5 Reliability analysis

**Fig. 19:**
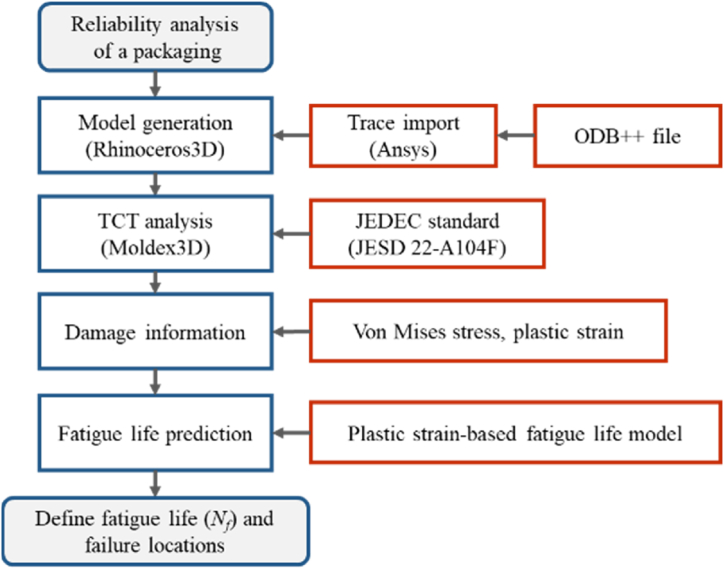
Reliability analysis process.

This section focuses on the reliability of the RDL and copper pillars in the VFBGA package. Fig. 19 shows the reliability analysis process. In the reliability analysis, trace import was used to establish the detailed RDL mesh model for a thermal cycling analysis. Then, the TCT simulation was conducted with process parameters from the JEDEC standard, and the stress distribution was observed to find out the location of potential failure. Finally, the fatigue life was calculated to predict the reliability of the product by substituting variable parameters and the results of the simulations into the fatigue model. In the reliability simulations, the use of an elastic material model did not account for the possibility of plastic strain because of the limitation of the simulated software.

### 5.1 Mesh model

**Fig. 20:**
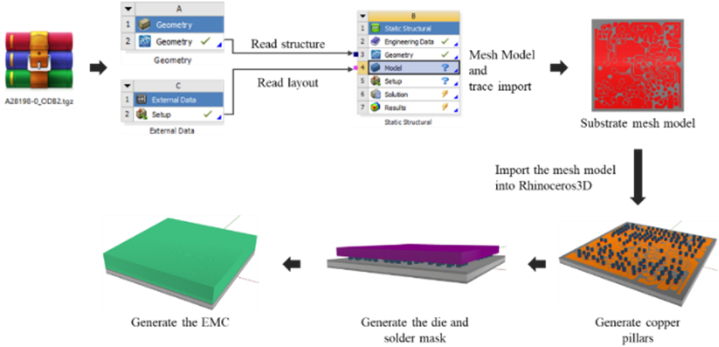
Schematic representation of the modeling process of a single packaging.

Because of the calculation limitations of the simulation facilities, the thermal cycle test analysis was performed through a single packaging model which has a single die in a package. The plane design of each layer structure inside a single unit substrate was provided by Advanced Semiconductor Engineering Inc. located in Kaohsiung, Taiwan. The modeling part was particularly detailed for vias, copper pillars, and the RDL distribution of the L1 and L2 mesh description. Considering reliability analysis requires predicting the failure locations of RDL traces and copper bumps, as well as establishing models for chips and EMC, trace import method was used for modeling. Fig. 20 shows the modeling process of single packaging.

### 5.2 Temperature settings of thermal cycle analysis

**Fig. 21:**
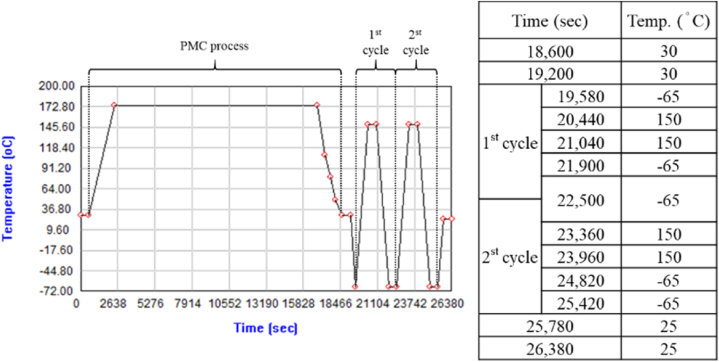
Simulated temperature curve and set parameters.

The temperature cycling conditions were based on the JESD22-A104F specification. The simulated temperature curve is shown in Fig. 21. As the thermal cycle analysis of a single package followed the PMC process of the strip, the temperature was set as 30 °C for 600 s to cool the packaging from the mold temperature of 185 °C before the start of the thermal cycle and then cool down to the initial temperature of the thermal cycle test condition, −65 °C, the cooling time was 380 s, calculated using the temperature change rate of 15 °C/min. In all, two thermal cycles were performed, and each cycle followed the conditions shown in Fig. 21 until the thermal cycle test was completed at 25,420 s, and the temperature increased to the room temperature of 25 °C. This temperature was maintained for 10 min to ensure that the packaging reached a uniform temperature.

### 5.3 Thermal cycle analysis results

**Fig. 22:**
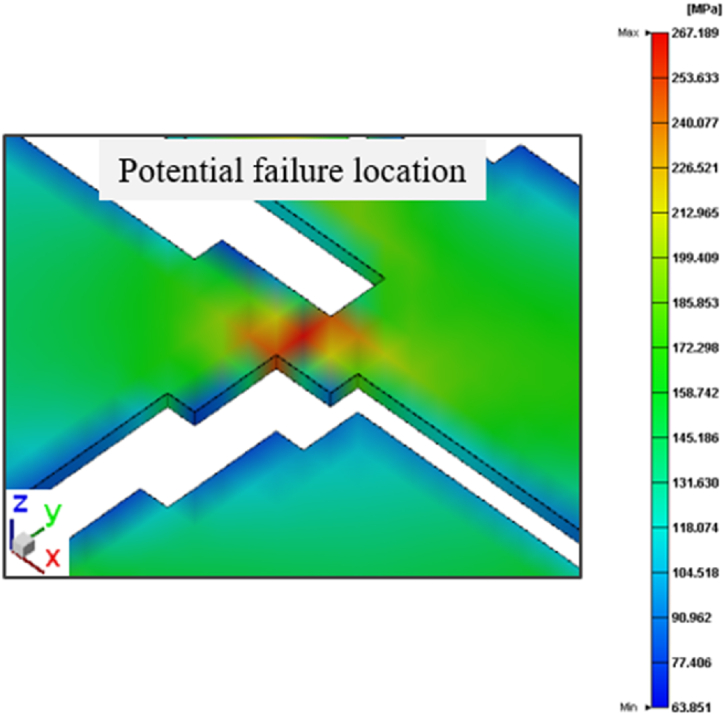
Potential failure location on RDL (L1).

From the Von Mises stress results of two thermal cycles, it was observed that the maximum value of the entire packaging mainly appeared in the RDL layer, so L1 and L2 were extracted at 19,200 s (30 °C), 23,960 s (150 °C), 25,420 s (−65 °C), and 26,380 s (25 °C). Finally, the local enlarged area shown in Fig. 22 was used as the potential failure location of RDL.

**Fig. 23:**
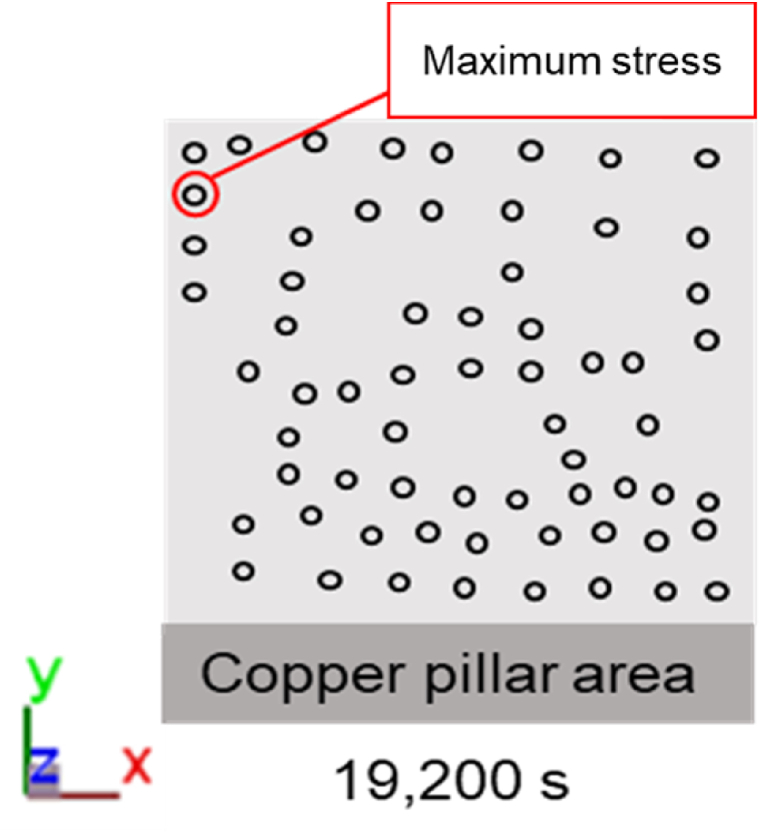
Maximum stress location of the copper pillars.

In addition to the RDL stress distribution, the copper pillars were one of the targets of the reliability study; the stress distribution was captured at 19,200 s (30 °C), 23,960 s (150 °C), 25,420 s (−65 °C), and 26,380 s (25 °C). Fig. 23 shows that the location with the maximum Von Mises stress value during the entire thermal cycle was the copper pillar in the upper-left corner of the unit packaging. Next, a stress analysis was conducted on the copper pillar at this location.

**Fig. 24:**
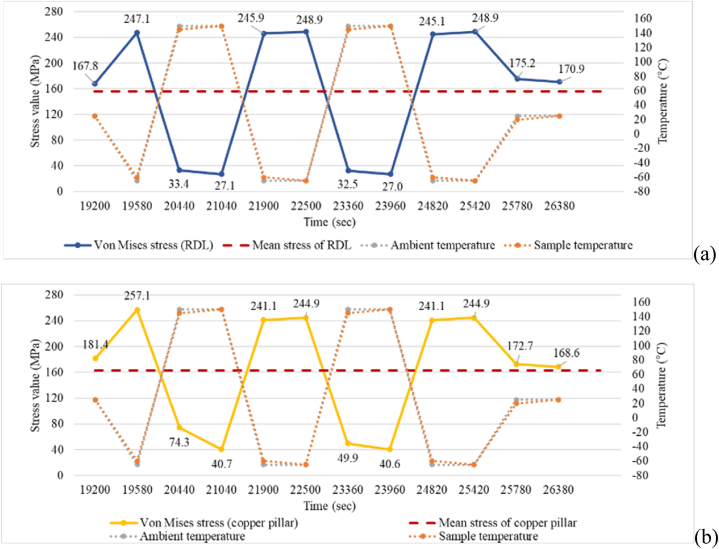
Von Mises stress and thermal cycle time relationship diagram: (a) maximum stress at the potential failure location of RDL, and (b) maximum stress at the potential failure location of copper pillars.

As shown in Fig. 24, both the RDL and the copper pillars exhibited the maximum stress values when the average packaging temperature reached −65 °C, which were 247.1 MPa and 257.1 MPa, respectively. The average stress (mean stress) of the RDL and the copper pillars was 156 MPa and 163 MPa, respectively. Since the goal of this study is to consider the effects of manufacturing process such as molding and post-mold cure processes on the reliability of a package, the residual stress due to these manufacturing process should be identified in this paper. Thus, it is considered in Fig. 24 that residual stress during processing is presented as mean stress in the thermal cycling test simulation. When the temperature varies, the stress will vary between the highest and lowest stress values. Table 3, Table 4 describe the variation of Von Mises stress over time for RDL and copper pillar, respectively.

**Table 3:** Von Mises stresses on RDL over time.

Time (s)	Stress Value (MPa)
19200	167.8
19580	247.1
20440	33.4
21040	27.1
21900	245.9
22500	248.9
23360	32.5
23960	27.0
24820	245.1
25420	248.9
25780	175.2
26380	170.9

**Table 4:** Von Mises stresses on copper pillar over time.

Time (s)	Stress Value (MPa)
19200	181.4
19580	257.1
20440	74.3
21040	40.7
21900	241.1
22500	244.9
23360	49.9
23960	40.6
24820	241.1
25420	244.9
25780	172.7
26380	168.6

### 5.4 Reliability calculation

After obtaining the information of the stress change with temperature in the thermal cycle analysis, the number of cycles of product failure was further predicted through a suitable fatigue model. For the metal materials of the RDL and the copper pillars, when the thermal stress exceeded the yield stress of the material, it caused a permanent plastic strain, which was the quantitative index for the fatigue life calculation.

The Ramberg–Osgood relation [34] was used to describe the relationship between elastic and plastic strain versus stress. The equation is as follows:

(16) $$\varepsilon_{total}=\varepsilon_{el}+\varepsilon_{p}$$

(17) $$\varepsilon_{total}=\frac{\sigma }{E}+\alpha \frac{\sigma_{y}}{E}{\left(\frac{\sigma }{\sigma_{y}}\right)}^{n‐1}$$

*ε*_*total*_: total strain; *ε*_*el*_, *ε*_*p*_: elastic and plastic strain; σ: equivalent stress; *E*: Young's modulus; *α*: material constant; *α*_*y*_: yield stress; *n*: hardening exponent. The material constant terms *α* and *n* are 3/7 and 3, respectively, referring to Wu [35]. Table 5 shows Young's modulus, yield stress, maximum value of equivalent stress at possible failure locations, and strain calculation results for the RDL and the copper pillars.

**Table 5:** Parameters and results for calculation of strain.

	*E* (GPa)	*σ*_*y*_ (MPa)	Equivalent stress (MPa)	Total strain range (%)	Plastic tensile strain range (%)
RDL (L1)	110	216	248.9	0.37	0.15
Copper pillar	110	100	257.1	0.49	0.26

According to the Coffin–Manson model calculation model used in the study of Che [27], the total strain (*ε*_*total*_) was substituted into the plastic strain (*ε*_*p*_) term of the original formula; the formula was as follows:

(18) $${N_{f}}^{m}\Delta \varepsilon_{total}=C_{f}$$

*N*_*f*_: fatigue life cycles; *m*: fatigue ductility exponent; Δ *ε*_*total*_: total strain; *C*_*f*_: ductility coefficient.

Table 6 lists the model parameters of the RDL and the copper pillars from Che [27], and the fatigue life values calculated using formula (18). The RDL and copper bumps were 9706 and 1689 cycles, respectively.

**Table 6:** Coffin–Manson model parameters and fatigue life calculation results.

Model parameters	RDL	Copper pillar
*m*	0.41	0.43
*C*_*f*_	0.16	0.12
Fatigue life prediction		
*N*_*f*_ (cycles)	9706	1689

**Fig. 25:**
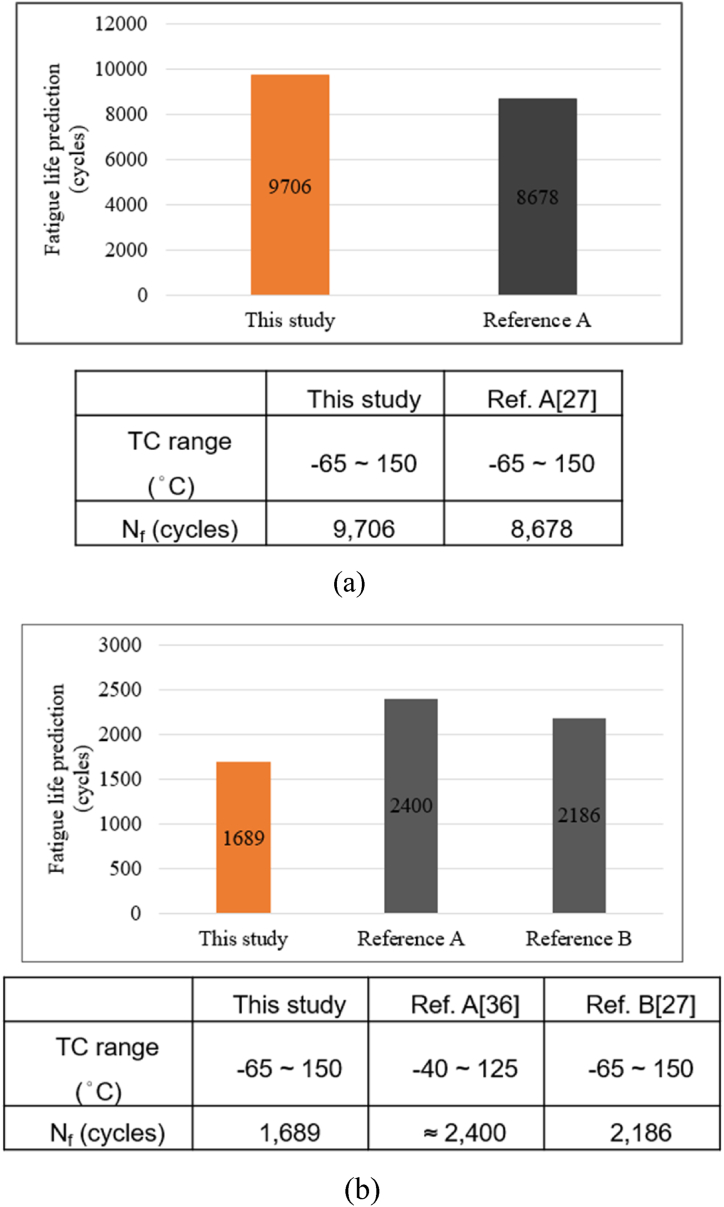
Comparison of fatigue life prediction results of (a) RDL and (b) copper pillars.

To confirm whether the predicted result is within a reasonable range, it will be compared with the results of relevant studies. From the comparison results shown in Fig. 25, the reliability cycle life numbers of the two potential failure locations of the RDL and the copper pillars in this study were similar to the results of other relevant studies [[27], [36]]. The differences were related to the size of the analysis model, the location of failure, and the difference in material properties (such as yield stress, tensile strength, and Young's modulus).

## 6 Conclusions

This study used the Moldex3D commercial simulation software to establish a simulation analysis process from the EMC filling process to determine the reliability for VFBGA strip packages, including EMC mold flow process, warpage, and residual stress prediction after demolding and PMC, and die sawing after the thermal cycle reliability analysis, the number of fatigue life cycle predictions and the potential failure locations of packaged products were finally proposed. Based on the *P–V–T–C* model and the generalized Maxwell's viscoelastic model, the simulated value of warpage after cooling to room temperature was 5.7 mm after filling EMC, while the predicted value of warpage after PMC was 5.0 mm, which was within the range of the actual measured value (3.0–4.8 mm) had a minimum error of approximately 3.9 %, and the overall warpage trend had a double egg yolk concave downward shape.

In the reliability analysis, the thermal cycle analysis results of the trace import model revealed that the potential failure locations were copper pillars and RDL. The maximum von Mises stresses of the copper pillar and RDL L1 at the lowest temperature (−65 °C) in the thermal cycle were 257.1 MPa and 248.9 MPa, respectively. According to the Coffin–Manson model, the potential fatigue life of the copper pillar and RDL L1 was 1689 and 9706 cycles, respectively.

## 7 Outlook

Due to the size limitation of mesh establishing for the RDL circuit, the equivalent method of volume percentage is the only option for establishing the RDL layer in the full strip. Find a more detailed method for mesh establishing on the circuit of RDL to make the warpage and stress results of the full strip closer to reality.

## CRediT authorship contribution statement

**Chin-Hsin Lo:** Writing – review & editing, Writing – original draft, Methodology, Formal analysis. **Te-Yuan Chang:** Writing – review & editing, Methodology, Formal analysis. **Ting-Yu Lee:** Writing – review & editing, Methodology, Formal analysis. **Sheng-Jye Hwang:** Writing – original draft, Supervision, Methodology.

## Declaration of competing interest

The authors declare the following financial interests/personal relationships which may be considered as potential competing interests:Sheng-Jye Hwang reports financial support was provided by Advanced Semiconductor Engineering Inc. If there are other authors, they declare that they have no known competing financial interests or personal relationships that could have appeared to influence the work reported in this paper.
